# Scarlet Fever

**Published:** 1840-11

**Authors:** 


### SCARLET FEVER.

It is worthy of being noted, that this foreigner seems, in fact, to have become naturalized among us—though it has not made a special "location." In past times it appeared as an occasional epidemic; but in these latter days it has been sojourning among our towns and neighborhoods,—now here and now there, sometimes mild, sometimes malignant, generally the former with as much quiet composure as one of our own veritable endemics. Such a gentle but protracted invasion would seem to afford many opportunities for studying its mode of dissemination, the means by which it destroys life, and the measures by which it may be successfully countervailed. We are sorry to find, however, that the profession are every where divided on these different points. Some cry out for—others against contagion: some stand up for acute inflammation and a liberal use of antiphlogistics—others stimulate throughout the whole course of the disease. Finally others do nothing. Each party has a certain number of facts to offer in support of their opinions, and seem little disposed to open their eyes to an examination of any others. In the midst of this great contrariety, but three truths are perceptible: first, that scarlatina *will* run its course; second that, when mild in character, the patient lives under *any* plan of treatment; third, that when malignant he dies *whatever* remedies may be employed. Such at least are our impressions, but we shall be most happy to publish evidences that the last is untrue. D.

